# Supplementary material for: Correction: The Post-Embargo Open Access Citation Advantage: It Exists (Probably), It's Modest (Usually), and the Rich Get Richer (of Course)
Source: PLoS One. 2016 Oct 21;11(10):e0165166. doi: 10.1371/journal.pone.0165166 (PMC5074514; doi:10.1371/journal.pone.0165166)
Supplement: S1 File — (PDF) [file pone.0165166.s001.pdf]

RESEARCH ARTICLE

The Post-Embargo Open Access Citation Advantage: It Exists (Probably), Its Modest (Usually), and the Rich Get Richer (of Course)

Jim Ottaviani*

Michigan Publishing, University Library, University of Michigan, Ann Arbor, Michigan, United States of America

* jim.ottaviani@umich.edu

OPEN ACCESS

Citation: Ottaviani J (2016) The Post-Embargo Open Access Citation Advantage: It Exists (Probably), Its Modest (Usually), and the Rich Get Richer (of Course). PLoS ONE 11(8): e0159614. doi:10.1371/journal.pone.0159614

Editor: Lutz Bornmann, GERMANY

Received: April 22, 2016

Accepted: July 6, 2016

Published: August 22, 2016

Copyright: © 2016 Jim Ottaviani. This is an open access article distributed under the terms of the Creative Commons Attribution License, which permits unrestricted use, distribution, and reproduction in any medium, provided the original author and source are credited.

Data Availability Statement: Data are available in anonymized form via Deep Blue repository (URL: deepblue.lib.umich.edu/data; DOI:10.7302/Z2KH0K8V).

Funding: This work was supported by the University of Michigan, University Library Research and Creative Projects Committee; Michigan Publishing. The funder had no role in study design, data collection and analysis, decision to publish, or preparation of the manuscript.

Competing Interests: The author has declared that no competing interests exist.

Abstract

Many studies show that open access (OA) articles—articles from scholarly journals made freely available to readers without requiring subscription fees—are downloaded, and presumably read, more often than closed access/subscription-only articles. Assertions that OA articles are also cited more often generate more controversy. Confounding factors (authors may self-select only the best articles to make OA; absence of an appropriate control group of non-OA articles with which to compare citation figures; conflation of pre-publication vs. published/publisher versions of articles, etc.) make demonstrating a real citation difference difficult. This study addresses those factors and shows that an open access citation advantage as high as 19% exists, even when articles are embargoed during some or all of their prime citation years. Not surprisingly, better (defined as above median) articles gain more when made OA.

Introduction

All things being equal, that an article made freely available ought to get downloaded more than a comparable article that costs money to access seems obvious. We mistrust the intuitively obvious, though, largely because all things are rarely equal and confounding factors are not always easy to intuit. Besides, more downloads (and presumably more readers) may not be sufficient motivation for authors to make their articles open access (OA); researchers want their work to have a measurable impact on their peers. They want to advance their field, boost their careers by increasing their likelihood of receiving grants, and improve their chances for promotion. . .preferably all three.

Citations are the coin of the impact realm, so a citation, not merely a download or readership, advantage is important to authors. A number of studies show that some kind of open access citation advantage (OACA) exists. For example, an oft-cited paper by Hajjem, Harnad and Gingras [1] found a 36–172% advantage, and the majority of studies (46/74) listed in [2] also report an OACA. Many of these same studies have also been challenged, though:

- One frequently expressed concern is that authors self-select only articles of higher than average quality to make OA, which would be expected to get more citations regardless of whether they were open ([3–7], e.g.).

- OA articles may have been previously available in working paper or pre-print versions that differ from their final published form. The resulting final publications may benefit from that early availability ([8–10], e.g.). Further, comparing the author's accepted manuscript for one article to the publisher's formatted and copy-edited version for another introduces a confounding variable in terms of quality, or at least perceived quality. A like-with-like comparison to determine whether an OACA truly exists is challenging in such scenarios.
- It can be difficult to find articles of any kind that have been open for long enough, after being previously closed, to show a meaningful effect on citation frequency [11].
- Finally, even if the above concerns are addressed, finding enough articles in a broad enough range of disciplines to draw a conclusion on an OACA has proved challenging. Studies in specific disciplines or single journals abound: 2/3 of the articles in [2] investigate citations and OA in only a single discipline, and over half of those studies use a small sample size or did not open articles for long. But even when convincing, such studies are prone to being dismissed as special cases, peculiar to the particular discipline or even sub-discipline.

One way to address these concerns would be to find an appropriate control group for a large sampling of OA articles. This too is difficult; every article is (or at least should be!) unique, and even so-called hybrid journals, which make some but not all articles OA, do not offer a straightforward means for comparison because of the self-selection problem, since they require authors to pay an additional publication fee to make them open from the outset.

Methodology

In Deep Blue <deepblue.lib.umich.edu/documents>, the University of Michigan's institutional repository service, we have the equivalent of a random sample of thousands of OA articles from thousands of journals. Each article has the following characteristics: Prior to a known date (ranging from 2006 onward) these articles, since they are the final published version, were only available by subscription. After that date, they became freely available via Deep Blue. Meanwhile, other articles from the same journal issue as the now-OA article continued to only be available to subscribers. None of the OA articles were self-selected; authors did not choose to deposit the articles in question in Deep Blue, since they were opened via blanket licensing agreements between the publishers and the library.

By comparing citations to subscriber-only/now-open (opened) articles with the corresponding subscriber-only/still-subscriber-only (closed) articles in that journal issue before and after availability in Deep Blue, we can determine what effect opening them may have had, i.e. a post-embargo OACA.

The sample began with a random selection of 3,850 papers—peer-reviewed and review articles only; bibliographies, book reviews, corrections, discussions, editorials, letters, notes, etc. were not considered—with original publication dates ranging from 1990 to 2013. These were matched with the 89,895 corresponding articles which remained closed, using the specific journal issue as a proxy for comparability of subject matter and quality. (It is an imperfect proxy, of course, but as noted above, in theory each article is unique, so an exact like-for-like comparison is not possible.) Using data from Thomson Reuters' Web of Science and Journal Citation Reports databases, we get actual citations and can calculate the expected values of citations to an article for each issue. (Some opened articles had no corresponding peer reviewed or review articles in that issue, and so were dropped from the sample. In issues where more than one opened article appeared, one was randomly selected to compare with the closed articles.) These are the important values to compare:

O_c = citations to opened article, while it was closed

O_o = citations to opened article, once opened

C_c = citations to other (closed) articles in that same issue, for the same period as O_c

C_o = citations to those same closed articles, for the same period as O_o

The null hypothesis (H_0) is straightforward:

$$H_0: \ \text{ActualCitations}_{\text{after opening}} - \text{ExpectedCitations}_{\text{after opening}} = 0$$

or

$$H_0: \ O_o - E = 0$$

In other words, assume that making an article OA has no effect and test that assumption. As an example of how a data point was calculated, if the article was opened in 2006 the numbers might look like this:

O_c = citations to opened article, through 2006 (i.e. citations to it while it was closed) = 20

O_o = citations to opened article, 2007–2014 (now OA) = 25

C_c = citations to other, closed articles in that same issue, through 2006 = 10

C_o = citations to other, closed articles, 2007–2014 = 15

To test H_0 we need to know how many citations to the opened article we would expect (E) if it behaved like the other articles in its issue that remained closed. In our example:

$$E = (C_o/C_c) * O_c = (15/10) * 20 = 30$$

so O_o—E = 25–30 = -5, meaning in this case the opened article had five fewer citations than expected.

Looking at all the articles in aggregate is straightforward and, as described below, doing so produces large OACA percentages, similar in magnitude to those previously reported. However, a more appropriate approach is to focus on article-by-article differences, calculated three ways: each opened article is compared to the mean and median article in the same journal issue which it appeared in and also to equivalent articles in that issue. Recognizing that other definitions are possible, we define "equivalent articles" as follows:

- If one or more articles had the same number of citations to the opened article while all were closed, use those articles;
- if not, but there were articles with citation counts during the closed period that bracket that of the opened article, use those;
- if the opened article had more, or fewer, citations than any other article, use the mean for the issue as an equivalent (this is conservative, since it will tend to maximize the effect of outliers among the closed articles).

Regardless of whether we use the median or the equivalent articles to calculate the expected value, the problem of dealing with never-cited articles comes up. (And it comes up often; in our sample the modal value for citations for all four groups of articles, O_c through C_o, was 0.) These zeroes were handled as follows:

If $C_c = 0$ for medians

$$E = \text{median } C_o - \text{median } C_c$$

for equivalents

$$E = \text{mean of equivalent } C_o\text{s} - \text{mean of equivalent } C_c\text{s}$$

Also, if $E < 0$, be conservative (and realistic) and use 0, as this minimizes differences when $O_c = 0$ and $O_o > 0$.

Analysis

As mentioned above, an aggregate calculation is straightforward: find the overall mean (or median) values and find the percentage difference between the ratios:

$$((O_o/O_c) - (C_o/C_c))/(C_o/C_c)$$

The mean values for the sample were $O_c = 17.57$, $O_o = 12.28$, $C_c = 16.59$, $C_o = 8.55$, yielding an OACA of 35.6%. The median values ($O_c = 6$, $O_o = 5$, $C_c = 7$, $C_o = 3$) produce an even more impressive OACA of 94.4%.

However, even leaving aside the large standard deviations (ranging from 24.96 to 51.41) of these aggregate values, we know their distributions are far from normal—again, the mode for all four values is zero, and no article can have less than zero citations—so an article-by-article calculation is more appropriate.

The differences between expected values and actual values were usually small. (For the means, $963/3850 \leq \pm 1$; for medians $963/3850 \leq \pm 1$, and; for equivalents $1133/3850 \leq \pm 1$.) This is not surprising, given the modes and the conservative treatment of the never-cited articles described above. Figs 1–3 plot these distributions.

Was there a significant difference, and can we reject H_0? Because the sample size is large, the t- and Z-distributions are reasonable approximations of each other, so applying the usual formulae for standard deviation to these distributions offers a quantitative measure of the difference between actual and expected values, and confidence intervals for them. These are as follows:

Overall (n = 3850)

$O_o—E_{mean} = -0.936$, $\sigma = 20.13$, $p < 0.005$

$O_o—E_{median} = 2.271$, $\sigma = 19.11$, $p < 0.0005$

$O_o—E_{equivalent} = 0.438$, $\sigma = 18.163$, $p < 0.10$

Above median (n = 1882)

$O_o—E_{mean} = 0.533$, $\sigma = 19.18$, $p < 0.25$

$O_o—E_{median} = 3.187$, $\sigma = 23.80$, $p < 0.0005$

$O_o—E_{equivalent} = 1.124$, $\sigma = 19.96$, $p < 0.01$

OACAs are typically presented as percentages; doing so and applying a 99% confidence level yields:

Overall (n = 3850)

$O_o—E_{mean} = -7.6\% \pm 6.8$

$O_o—E_{median} = 18.5\% \pm 6.5$

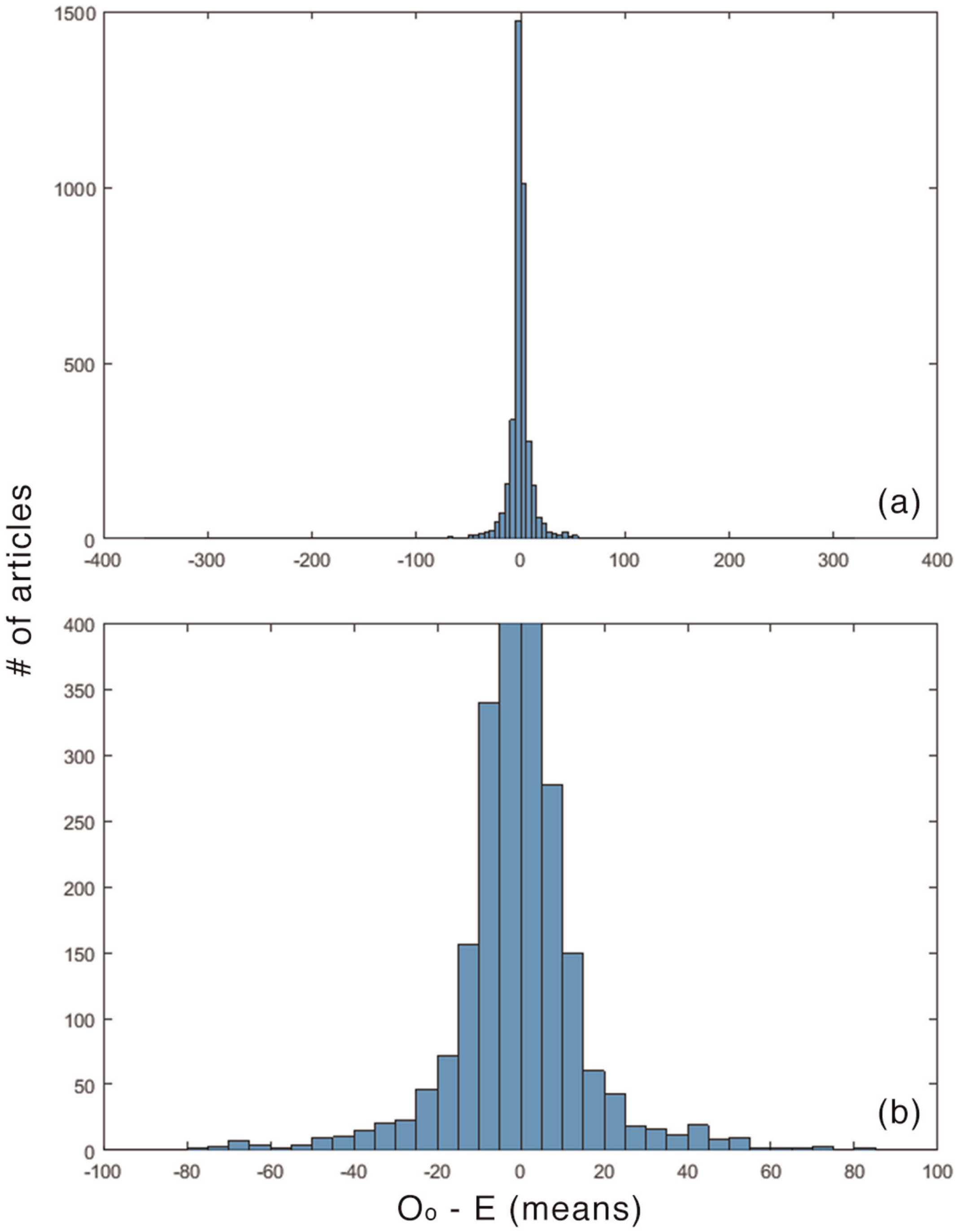

Fig 1. O_o—E for means. (a) overall (b) detail.

doi:10.1371/journal.pone.0159614.g001

O_o—$E_{equivalent}$ = 3.6% ± 6.5

Above median (n = 1882)

O_o—E_{mean} = 3.2% ± 7.1

O_o—E_{median} = 19.3% ± 10.6

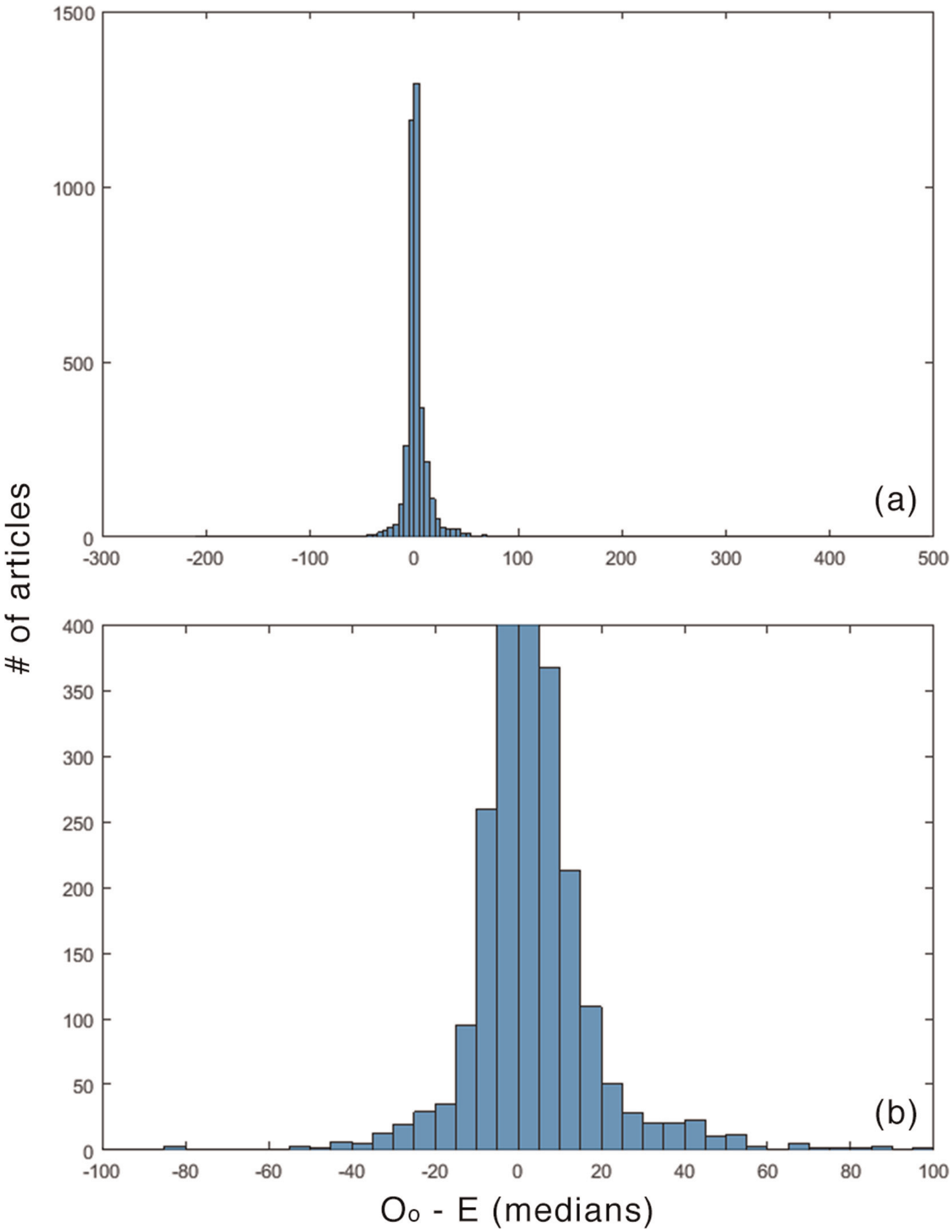

Fig 2. O_o—E for medians. (a) overall (b) detail.

doi:10.1371/journal.pone.0159614.g002

O_o—$E_{equivalent}$ = 6.8% ± 7.7

As a refinement, we can define an equivalent article more precisely by limiting the difference between O_c and C_c to ≤ 1%, (i.e., if citations to an opened article and its closed equivalent(s) differ by more than 1% during the period when both were closed, discard them since they were not actually similar enough). Doing so gives the following:

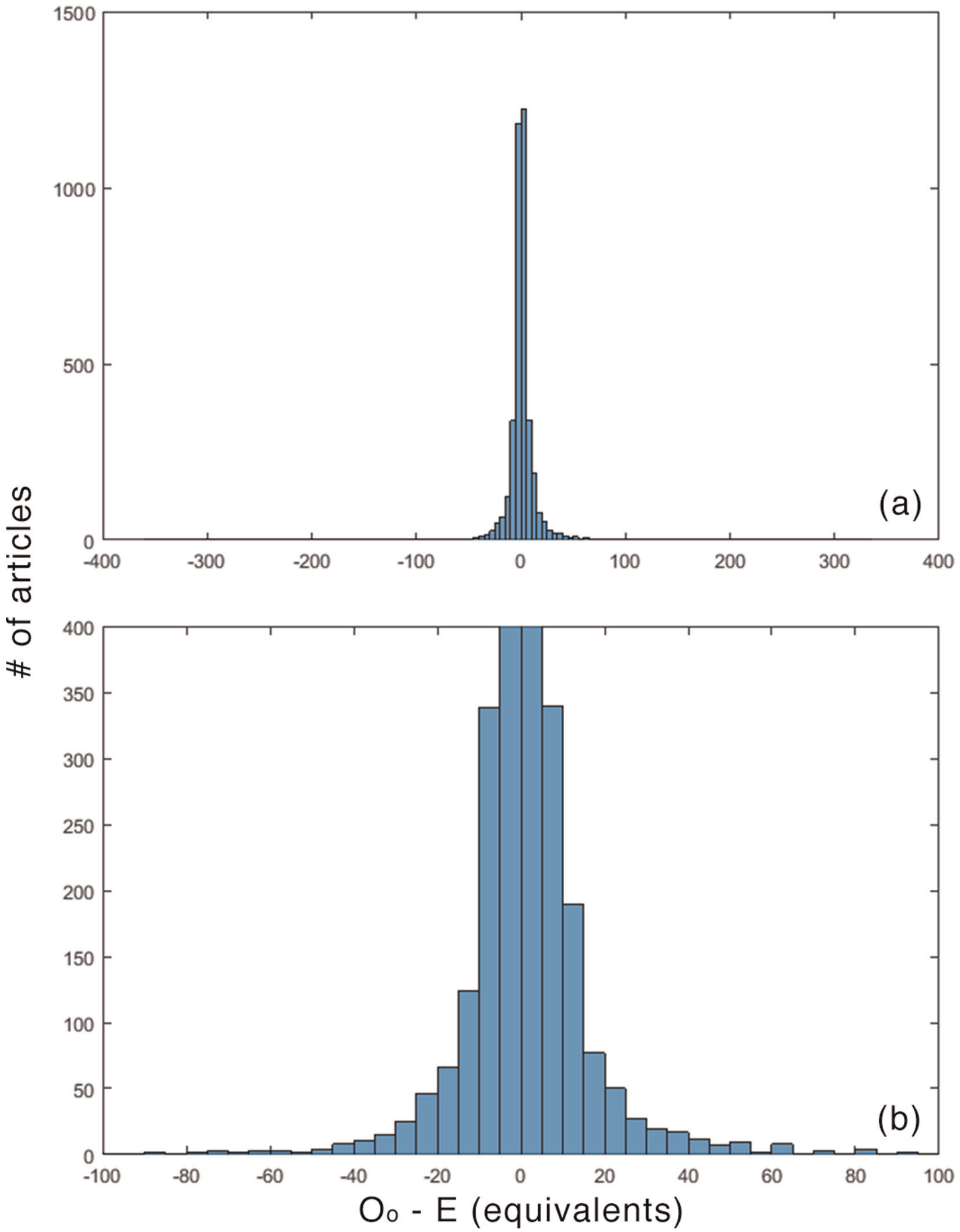

Fig 3. O_o—E for equivalents. (a) overall (b) detail.

doi:10.1371/journal.pone.0159614.g003

Overall (n = 2231, where $0.99 \leq O_c/C_c \leq 1.01$)

O_o—$E_{closest\ equivalent} = 0.927$, $\sigma = 10.209$, $p < 0.0005$

O_o—$E_{closest\ equivalent} = 10.7\% \pm 6.4$

Above median (n = 729)

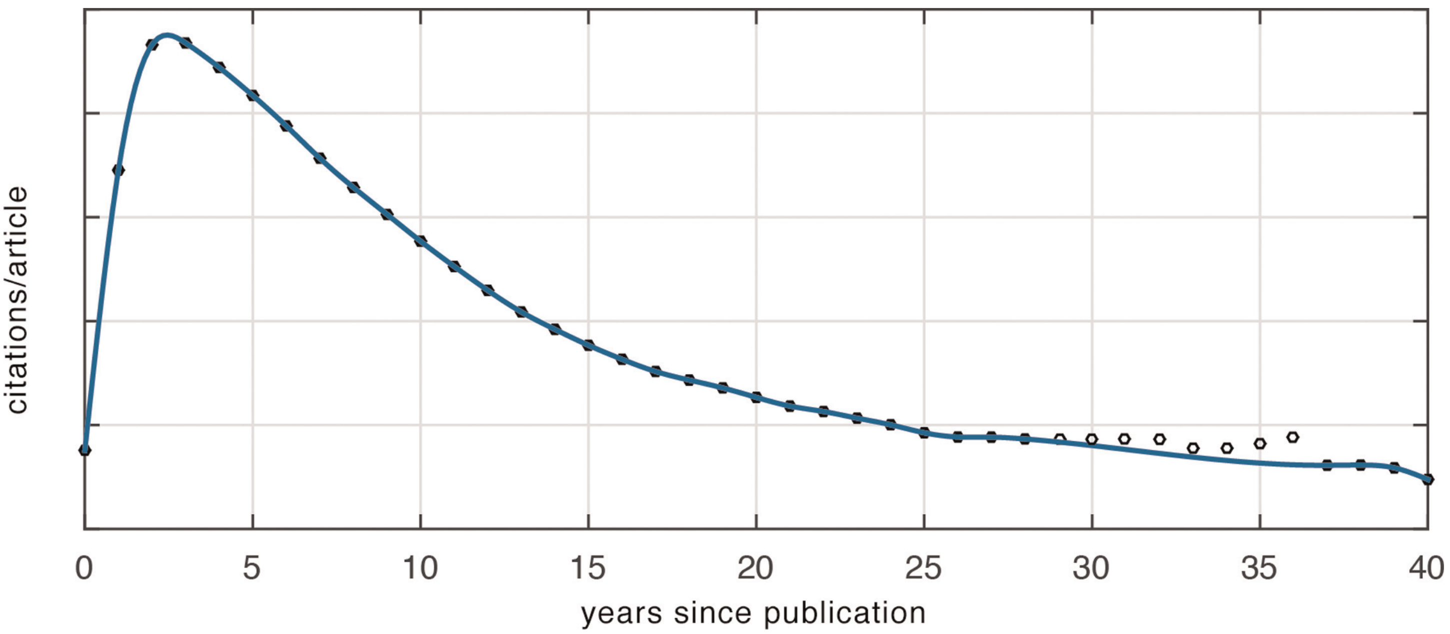

Fig 4. U-M citation curve, 1975–2013.

doi:10.1371/journal.pone.0159614.g004

$O_o—E_{closest\ equivalent} = 0.580$, $\sigma = 10.412$, $p < 0.10$

$O_o—E_{closest\ equivalent} = 6.0\% \pm 10.3$

Since the majority of articles in the overall sample were older (82% were more than five years old and 61% were more than ten years old), per Fig 4, most will have been past their prime citation years.

So, a further refinement is possible by repeating the analysis for only the newest articles (from publication to eight years old), and limiting those considered to ones that were closed less than 85% of their lifespan. In this case the aggregate OACA for the mean becomes even larger: 665.9%. (Because the median O_c is zero, the corresponding result for medians is indeterminate.) However, the results for the granular analysis for this smaller sample are more equivocal:

Overall (n = 416)

$O_o—E_{mean} = -8.096$, $\sigma = 37.68$, $p < 0.0005$ (i.e., -46.7% ± 27.5)

$O_o—E_{median} = 3.342$, $\sigma = 24.12$, $p < 0.005$ (19.3% ± 17.6)

$O_o—E_{equivalent} = -1.827$, $\sigma = 30.30$, $p < 0.25$ (-10.5% ± 22.1)

Above median (n = 92)

$O_o—E_{mean} = -24.10$, $\sigma = 60.93$, $p < 0.0005$ (-88.8% ± 60.3)

$O_o—E_{median} = 2.544$, $\sigma = 44.19$, $p < 0.40$ (9.4% ± 43.7)

$O_o—E_{equivalent} = -15.10$, $\sigma = 56.84$, $p < 0.01$ (-55.6% ± 56.2)

Discussion

We cannot draw conclusions—at least with high confidence—regarding the existence or absence of an OACA in every scenario; the p-values are too large for $O_o—E_{equivalent}$ (overall),

O_o—E_{mean} (above median), O_o—$E_{closest\ equivalent}$ (above median), and O_o—$E_{equivalent}$ (overall) and O_o—E_{median} (above median) for the newest articles to be confident in those values. Two things are clear, though.

First, in every case the overall sample the above median articles show a greater gain than the rest (3.2% > -7.6%, 19.3% > 18.5%, and 6.8% > 3.6%). So, in the long run better articles gain more citations than expected by being made OA, adding weight to the results reported by Gargouri et al. [3] (This does not appear to hold for the closest equivalents, though their high p-values make any conclusion about a gain or loss suspect.) Just like the O_o—E_{mean} value, where we find an open access disadvantage, these data appear intent on preventing us from making blanket statements.

Second, an OACA appears to exist for all but one case (opened articles compared to all other articles in the issue), but the confidence intervals are relatively small only in the case where we compare opened articles to their issue's medians.

It is arguable that the median is a better control group than the mean, since its use reduces the influence of outliers, and can correct for a potential bias in the sample as well: the opened articles come from one institution, and one that enjoys a good reputation for scholarship. So it is possible that, given that reputation, articles on the borderline of acceptance get more of a benefit of the doubt during the peer review process. Note, however, that the mean for O_c was 6% more than C_c, while the median was 14% less, so an acceptance bias might be argued either way.

Taken together, these somewhat equivocal results lead to a short discussion of the limitations of the data, which are available in anonymized form via Deep Blue <deepblue.lib.umich.edu/data; doi:10.7302/Z2KH0K8V>.

- Though it was not possible here, it is clear that a multi-institution sample would be ideal, as would a sample not dominated by physical science, health science, and engineering articles (92% of the total).
- As discussed above, the age of the articles available for study is an additional factor, as the majority of those studied were past their peak citation years. The results for the medians in the smaller sample of more recently published articles mirror the broader analysis, but there appears to be a negative effect on the means. When coupled with the small positive OACA found for the larger sample, one possibility is that OA mainly extends the shelf-life of research. Other explanations are possible, of course, but a larger sample of articles—still young enough themselves, and closed for a short enough time—is needed to demonstrate the true size of an OACA during an article's prime.
- With regards to articles being closed for a short enough time, given the evidence that any embargo reduces citations [10], the ideal would be no time at all. The articles in this sample were all embargoed during some or all of their prime citation years, so they may not have achieved their full OA potential.
- The citation counts here are probably conservative. Because the data from Web of Science is only available on a yearly basis, citations to an opened article (O_o) will always be under-counted and citations to that same article while closed (O_c) will be over-counted by some unknown amount. This may not be offset by using a more fine-grained approach for the corresponding articles that remained closed during that time.
- Context also matters. While OA repositories are typically well-indexed, making their contents easy to find via e.g. Google searches, opening an article in an institutional repository is not the same as opening it within the context of the journal itself or via a discipline-specific

repository. Making an article OA in context(s) more researchers consider a destination for conducting a literature search would likely produce more citation activity.

There are no doubt others, and despite these limitations, the data suggest additional angles from which to explore the effects of making articles OA. Some articles had only their metadata indexed by Google et al., while others had searchable full text as well. Looking at specific disciplines, the effect of journal impact factor, and changing the definition of what an equivalent (or closest equivalent) article come immediately to mind as additional avenues worth exploring.

Conclusion

Removing self-selection bias, considering only published versions of articles, assuring that articles were open for a long enough time to allow for meaningful citation patterns to emerge, and working with a large sample address the main weaknesses in previous attempts to show an OACA.

When treating all the articles in aggregate, this study produced an OACA of comparable magnitude to previous studies. That large advantage shrinks when articles are treated individually and compared to close equivalents, but it doesn't disappear. This may be because outliers skew the results (the rich can always get richer, but there's a lower limit to how poorly an article can be cited), or it may be that the best we can do with the data we have is to produce boundary values for the OACA. So, even though effects found here are more modest than reported elsewhere, given the conservative treatments of the data and when viewed in conjunction with other OACA studies already done [2,12], the results lend support to the existence of a real, measurable, open access citation advantage with a lower bound of approximately 20%; absent a large body of published articles available as OA from the moment of publication onward it may be difficult to say more. The publishing environment we have right now can not provide such a data set, and will not do so in the foreseeable future. The closest we have today are hybrid journals, but their OA articles are not only self-selected by authors (presumably because of higher than normal confidence in their quality), but by authors who have the ability to pay to assure immediate OA, doubling down on that selection bias.

It is in authors' best interest to create a more open environment. Combining the results of treating each article individually with the aggregate numbers for mean-to-mean and median-to-median comparisons, we see that when an article benefits from being OA, it benefits a lot. In other words, where there is an OACA it is large relative to the cases where the effect of OA is zero. Judging by the difference found between the smaller group of newer articles and the overall analysis, it's plausible that OA also extends an article's impact further into the long tail of the citation curve. And the better the author, the stronger the interest in open access, since, as shown by the analysis of above-median articles, OA (unsurprisingly) benefits better articles the most.

Acknowledgments

Many people helped me start, and complete, this study. Special thanks go to the Research and Creative Projects Committee at the University of Michigan Library for funding a pilot study; Charles Watkinson and Gabriela Beres for funding assistance for the full study; Timothy Otto, Steven Mobley, and their team at Thomson Reuters for their work to provide the raw data, and for their permission to make it available to others in an anonymized form; and Corey Powell and the staff at the Center for Statistical Consultation and Research for their assistance in analyzing the data.

Author Contributions

Conceived and designed the experiments: JO.

Performed the experiments: JO.

Analyzed the data: JO.

Wrote the paper: JO.

References

1. Hajjem C, Harnad S, Gingras Y. Ten-Year Cross-Disciplinary Comparison of the Growth of Open Access and How it Increases Research Citation Impact. 2006:[10 p.]. Available: http://arxiv.org/abs/cs/0606079 (an earlier version was published in IEEE Data Engineering Bulletin. 2005 December;28(4):[8 p.]. Available: http://sites.computer.org/debull/A05dec/hajjem.pdf).
2. SPARC Europe [Internet]. [cited 2016 April 11]. The Open Access Citation Advantage: List of studies and results to date. [about 40 screens]. Available: http://sparceurope.org/oaca_table/.
3. Gargouri Y, Hajjem C, Larivière V, Gingras Y, Carr L, Brody T, et al. (2010) "Self-Selected or Mandated, Open Access Increases Citation Impact for Higher Quality Research. PLOS ONE. 2010 October 18; 5 (10):e13636. doi: 10.1371/journal.pone.0013636 PMID: 20976155
4. Davis P. Open access, readership, citations: a randomized controlled trial of scientific journal publishing. The FASEB Journal (Journal of the Federation of American Societies for Experimental Biology). 2011 Mar 30:[6 p.]. doi: 10.1096/fj.11-183988
5. Gaulé P, Maystre N. Getting cited: Does open access help? Research Policy. 2011 December; 40 (10):1332–1338. doi: 10.1016/j.respol.2011.05.025
6. Xia J, Nakanishi K. Self-Selection and the Citation Advantage of Open Access Articles. Online Information Review, 2012; 36(1):40–51. doi: 10.1108/14684521211206953
7. McCabe MJ, Snyder CM. Does Online Availability Increase Citations? Theory and Evidence from a Panel of Economics and Business Journals. Social Science Research Network (SSRN). 2013 March 14:[50 p.]. doi: 10.2139/ssrn.1746243
8. Moed HF. The effect of 'Open Access' upon citation impact: An analysis of ArXiv's Condensed Matter Section. Journal of the American Society for Information Science and Technology. 2007 August 30; 58 (13):2145–2156. doi: 10.1002/asi.20663
9. Moed HF. The Effect of Open Access upon Citation Impact, Editors' Update. 2012 March 23 [cited 2016 April 11];[about 1 p.]. Available: http://www.elsevier.com/editors-update/story/access/the-effect-of-open-access-upon-citation-impact.
10. Gentil-Beccot A, Mele S, Brooks TC. Citing and reading behaviours in high-energy physics. Scientometrics. 2009; 84(2):345–355. doi: 10.1007/s11192-009-0111-1
11. Davis PM, Lewenstein BV, Simon DH, Booth JG, Connolly MJL. Open access publishing, article downloads, and citations: randomised controlled trial. BMJ 2008; 337:a568 [6p]. doi: 10.1136/bmj.a568 PMID: 18669565
12. The Open Citation Project [Internet]. [last updated 03/09/2009 12:42:07; cited 2016 April 11]. The effect of open access and downloads ('hits') on citation impact: a bibliography of studies. [about 1 screen]. Available: http://opcit.eprints.org/oacitation-biblio.html.
